# Dr. Beverley Stewart Ringer

**Published:** 1911-04-29

**Authors:** 


### OBITUARY.

Dr. Beverley Stewart Ringer has died at Stockbridge, Hants, at the age of sixty-six. He was formerly medical officer at the Chinese Hospital and H.B.M. Consulate, and the Chinese I.M. Customs at Amoy, China, and more recently at Canton. Dr. Ringer was the discoverer in Formosa of a parasitic worm known as *Distoma Ringeri*. He took his M.R.C.S. Eng. in 1869 and his L.S.A. Lond. in 1870, and in 1891 graduated M.D. at the University of Durham.

